# Innate immune genes of the chicken MHC and related regions

**DOI:** 10.1007/s00251-021-01229-2

**Published:** 2021-10-26

**Authors:** Jim Kaufman

**Affiliations:** Institute for Immunology and Infection Research, Ashworth Laboratories, University of Edinburgh, Charlotte Auerbach Road, Edinburgh, EH9 3FL UK

**Keywords:** Avian, B locus, Y locus, PRY-SPRY, B30.2

## Abstract

Compared to the major histocompatibility complex (MHC) of typical mammals, the chicken BF/BL region is small and simple, with most of the genes playing central roles in the adaptive immune response. However, some genes of the chicken MHC are almost certainly involved in innate immunity, such as the complement component C4 and the lectin-like receptor/ligand gene pair BNK and Blec. The poorly expressed classical class I molecule BF1 is known to be recognised by natural killer (NK) cells and, analogous to mammalian immune responses, the classical class I molecules BF1 and BF2, the CD1 homologs and the butyrophilin homologs called BG may be recognised by adaptive immune lymphocytes with semi-invariant receptors in a so-called adaptate manner. Moreover, the TRIM and BG regions next to the chicken MHC, along with the genetically unlinked Y and olfactory/scavenger receptor regions on the same chromosome, have multigene families almost certainly involved in innate and adaptate responses. On this chicken microchromosome, the simplicity of the adaptive immune gene systems contrasts with the complexity of the gene systems potentially involved in innate immunity.

## Introduction

The major histocompatibility complex (MHC) is the genetic region that determines rapid graft rejection, due to the highly polymorphic classical class I and class II molecules that play central roles in the adaptive immune system by presentation of peptides to T cells, as well as being important in innate immunity as ligands for natural killer (NK) cells (Kaufman 2018a). The MHC of typical mammals is a large genomic region with hundreds of genes, among which a few encode classical MHC molecules along with many genes that have other functions (Knight and Trowsdale 2013). By contrast, the chicken MHC is a small and simple region, expressing a single class I molecule and a single class II molecule at high levels, whose properties can determine the immune response (Kaufman 2018b, Kaufman et al. 1999a).

**Fig. 1:**
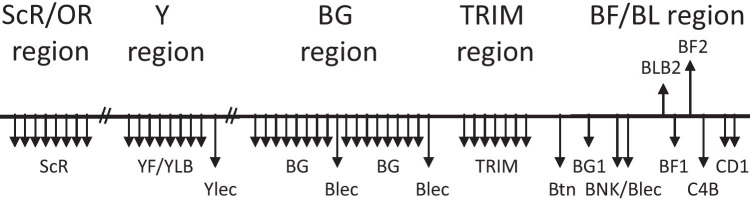
A schematic diagram to indicate the genes in the chicken MHC and adjacent chromosomal regions that are likely to be involved in innate or adaptate immune responses (downward-pointing arrows underneath the line depicting the genomic sequence), in comparison to the classical class I gene BF2 and the classical class II B gene BLB2 involved in the adaptive response (upward-pointing arrows above the line). To be clear, some multigene families have copy number variation, so the exact number of genes is not implied in this diagram. There are genes that are involved in supplying peptides to the class I and class II molecules that are not depicted

The chicken MHC is known for strong associations with resistance and susceptibility to economically important pathogens (Kaufman 2013, Miller and Taylor 2016), and so it perhaps is not too surprising that the focus of most chicken MHC research has been on the genes important for adaptive immunity. However, there are some genes in the chicken MHC which are likely to be important for innate immunity and/or for recognition by cells of the adaptive immune system with semi-invariant receptors (called by some “adaptate responses”, Hayday 2019; Hayday and Vantourout 2020). Moreover, the chicken MHC is embedded in a larger region and on the same chromosome with other genomic regions, and some of the genes in these regions may also be important for innate or adaptate immunity. This brief review will describe some aspects of these genes which have been neglected up to now, contrasting the complexity of the various innate systems with the simplicity of the adaptive immune system genes on this chromosome (Fig. 1).

## The chicken MHC, the B locus and the Y locus

**Fig. 2:**
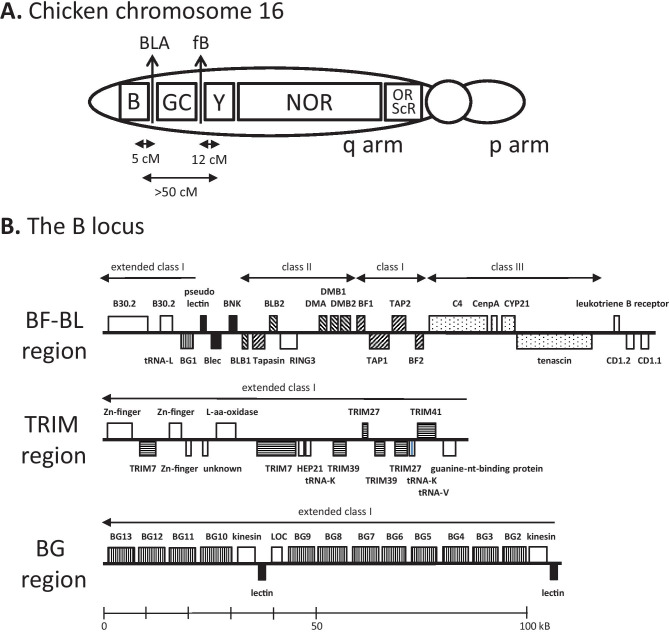
Organisation of regions on chicken chromosome 16, as currently understood. **A** Depiction of chromosome 16, based on analysis by FISH, radiation hybrids, genetics, southern blotting and sequencing. B, B locus; GC, G + C-rich region of PO1 repeats; Y, Rfp-Y region; NOR, nucleolar organiser region; BLA, class II A gene; fB, factor B gene; ORs, olfactory receptor genes; SRCRs, scavenger receptor with cysteine repeat genes. Double-headed arrows indicate recombination frequencies between B and BLA, fB and Rfp-Y, and B and Rfp-Y. **B** Region of the B locus currently sequenced, including the BF-BL region, the TRIM region and the BG region. Genes represented by boxes. Rising and falling stripes indicate genes of the classical class I and class II presentation system, respectively; stippled indicate class II region genes; black indicates lectin-like genes and pseudogenes; horizontal stripes indicate TRIM family genes; vertical stripes indicate BG genes. Names of genes above indicate transcription from left to right, below indicate transcription from right to left. (Figure modified from Kaufman 2013)

Altogether, the genomic regions on the long arm of chromosome 16 are understood in different levels of detail. These levels vary from mapping and cytogenetics to detailed molecular sequence (Fig. 2).

The chicken MHC is embedded in a larger historical genetic region called the B locus (or sometimes, the B complex), which was originally described based on the reactivity of alloantisera raised against blood cells and assessed by reactivity to erythrocytes (reviewed in Afrache et al. 2020). By a variety of methods, it was determined that two genetic loci separated by some level of recombination were involved, the BG region and the BF/BL region (Pink et al. 1977; Simonsen et al. 1982; Ziegler and Pink 1976). The BG antigens, whose closest relatives are the butyrophilins, were originally thought to be erythrocyte antigens, until it was shown that they are found on other cells of the hemopoietic lineage (including lymphoid and myeloid cells) as well as epithelial cells (Miller et al. 1990; Salomonsen et al. 1991, 2014). The BF antigens, originally described on erythrocytes and lymphocytes, are the chicken classical class I molecules. The BL antigens, originally described on B cells, are the chicken classical class II molecules. The B locus was found to determine resistance to the tumours induced by Marek’s disease virus (an oncogenic herpesvirus), with recombinants mapping the response to the BF/BL region (Briles et al. 1977, 1983; Plachy et al. 1992). The B locus was shown to be located on a microchromosome (now numbered as chromosome 16) along with the nucleolar organiser region (NOR) that contains many ribosomal RNA (rRNA) genes (Bloom and Bacon 1985).

The first step in understanding the B locus at the molecular level was through cosmids isolated by Francois Guillemot in the lab of Charles Auffray, who defined four cosmid clusters from the B12 haplotype, one of which is now known to cover much of the BF-BL region (Guillemot et al. 1988). When cosmids from this cluster were sequenced, it became clear that they constituted the BF/BL region which was the chicken MHC, defined as the genomic region responsible for rapid graft rejection. Compared to the MHC of typical mammals, this region was small and simple, containing a few polymorphic classical class I and class II B genes, polymorphic genes involved as in peptide loading and a single C4 gene, so that the BF-BL region was dubbed a “minimal essential MHC” (Kaufman et al. 1995, 1999a, b).

The other three cosmids, originally thought to be part of the MHC, were eventually understood to be derived from the Rfp-Y region that was discovered by Marcia Miller and colleagues based on restriction fragment polymorphism (Rfp) with BF gene probes (Briles et al. 1993; Miller et al. 1994, 1996). These Rfp-Y cosmids contained class I sequences related to the classical BF genes as well as class II B sequences related to the classical BLB genes (Miller et al. 1994; Zoorob et al. 1993); later, lectin-like genes related to Blec genes were described as well (Rogers et al. 2003). The Y region was found to have moderate effects on transplantation (at the level of minor histocompatibility antigens in mammals) and on responses to Rous sarcomas (LePage et al. 2000; Thoraval et al. 2003). Despite being on the same chromosome, the BF/BL region and the Y region were separated by sufficient recombination as to segregate entirely independently. Originally, this separation was attributed to the NOR, but eventually cytogenetics showed that the two loci are separated by a region of repeats, perhaps originally described as sub-telomeric repeats (Delany et al. 2009).

Additional cloning and sequencing filled out some of the regions on either side of the BF/BL region. On one side, additional genes joined the C4 gene as the class III region of the MHC, followed by a pair of CD1 genes, with CD1 genes in mammals found in an MHC paralogous region on a chromosome other than the MHC (Maruoka et al. 2005; Miller et al. 2005; Salomonsen et al. 2005). On the other side, cosmids and BACs were used to define a region with tripartite motif (TRIM) and several other kinds of genes, followed by the BG region which contained primarily BG genes along with a few lectin-like genes related to Blec genes (Ruby et al 2005; Salomonsen et al. 2014; Shiina et al. 2007). Some TRIM genes are found in and around the MHC of mammals, with other TRIM genes located elsewhere. Two other genes known to be in the MHC of mammals had been cloned as chicken cDNAs and used to map their location: the class II A gene known as BLA was found roughly 5 cM from the BF/BL region, and the complement component factor B was located roughly 12 cM from the Y region (Kaufman et al. 1999; Koch et al. 1986; Salomonsen et al. 2003). Neither of these genes has yet been identified in any genomic sequence. Finally, BAC cloning, cytogenetics and sequencing were used to locate two other groups of genes, olfactory receptors and scavenger receptors (along with other genes), the first found in the class I regions and the second not on MHC chromosomes in mammals (Miller et al 2014; Warren et al 2017).

It is perhaps helpful to describe a schism in the names given to these various regions. The original paper reporting the sequence of the BF/BL region (Kaufman et al. 1999a) described it as the chicken MHC, due to the presence of the polymorphic classical class I and class II genes responsible for graft rejection as well as a variety of in vivo and in vitro assays emblematic of the MHC of mammals. However, the organisation of this MHC was arranged differently than in mammals, with the class III region on the outside and the TAP genes in between the two class I genes, an organisation suggested to be ancestral. Moreover, some additional genes were present, including a pair of lectin-like genes (BNK and Blec) whose orthologs are located in the natural killer complex (NKC), sometimes considered to be another MHC paralogous region. CD1 genes, which in mammals are also in an MHC paralogous region, were later found outside of the chicken class III region (Maruoka et al. 2005; Miller et al. 2005; Salomonsen et al. 2005). However, many genes expected from the MHC of mammals were missing. Following up some examples, the C2 gene of the complement cascade, which is found next to factor B and C4 genes in class III region of mammals, was found on chicken chromosome 20 (Kaufman 2013). A more recent example is the TNF (or TNFα) gene, which in mammals is found in the class III region, located on an unplaced scaffold, which in a crow genome is surrounded by many of the same genes as mammals (Rohde et al. 2018). Early on, this led to the notion that the genes missing from the BF/BL region were either absent or moved elsewhere in the chicken genome (Kaufman et al. 1999a, b).

In contrast to this view based on function, some colleagues more influenced by genomics consider that all regions on chicken chromosome 16 with genes associated with any MHC of mammals should rightly be called MHC regions (Miller and Taylor 2016; Miller et al. 2004). Thus, the region around the BF-BL region has been called MHC-B, while regions around the Rfp-Y region have been named MHC-Y. Included in MHC-B and MHC-Y would be those genes which in mammals are located on different chromosomes, such as the lectin-like genes BNK and Blec. Thus far, no comment has been made for those chicken regions with orthologs that in mammals are in the MHC, but in chickens are in different chromosomes; the region on chromosome 20 with C2 gene(s) could be called MHC-C2 in this schema. The differences in nomenclature based on genomics and on function are not of any ultimate importance, although the functional view is that the MHC is defined by the iconic gene systems present, with all other genes being part of the MHC syntenic region in which different genes come and go over long periods of evolutionary history (Kaufman 2018a, b).

## Innate immunity genes of the BF/BL region

Most of the genes in the chicken MHC are involved in adaptive immunity. The genes of the classical class I system include the classical class I genes BF1 and BF2, the genes for the transporter associated with antigen presentation TAP1 and TAP2, and the bespoke class I chaperone and peptide editor gene tapasin (also called, TAP binding protein or TAPBP) (Kaufman 2015a, b, 2018b, 1999b). The genes of the class II system include the classical class II B genes BLB1 and BLB2, and the bespoke class II chaperone and peptide editor genes DMA, DMB1 and DMB2 (Parker and Kaufman 2017; Kaufman et al. 1999b). Despite not knowing the exact location for the classical class II A gene BLA, whose nearly monomorphic gene product BLα forms a heterodimer with polymorphic BLβ chains, it has sometimes been considered part of the chicken MHC. Another way to think of it is the same as β_2_-microglobulin (β_2_m) gene, found outside of the MHC in all vertebrates except cartilaginous fish, encoding the monomorphic partner of polymorphic class I heavy (α) chains; in this sense, β_2_m and BLA genes encode average best fits for whatever partner chains are expressed (Kaufman 1999). CD1 heavy chains also bind β_2_m to form CD1 molecules in both mammals and chickens (Pickel et al. 1990; Salomonsen et al. 2005), which present lipids to T cells as part of the adaptive immune system.

There are other genes in the chicken MHC that are not obviously involved in immunity, such as those that encode the somewhat mysterious serine/threonine kinase called BRD2 (or sometimes RING3) involved in epigenetics (Denis 2010; Thorpe et al. 1996), the enzyme steroid hydroxylase which is a cytochrome P450 monooxygenase with a major role in adrenal steroidogenesis (Haider et al. 2013), the extracellular matrix glycoprotein tenascin B (TNXB) involved in collagen binding and the centromere protein A (CenpA) which is a Histone H3–like nucleosomal protein involved in the kinetochore (which does seem to be involved in autoimmunity, Vakdivia et al. 2009). However, such genes can be involved in resistance and susceptibility to pathogens, such as the interaction of chicken BRD2 with Newcastle disease virus replication (Duan et al. 2020), and some tenascin family members are involved in binding chemokines (Orend and Tucker 2021).

A third group of chicken MHC genes are (potentially) involved with innate immunity. One gene next to the CD1 genes encodes a leukotriene B4 receptor for potent chemoattractants involved in inflammation (Saeki and Yokomizo 2017), some genes are involved with NK cells, one gene with the complement cascade and a couple of genes potentially with γδ T cells.

Studies of NK cells in mammals have shown that many viruses evade cytotoxic T lymphocytes (CTLs) by downregulating class I molecules in one fashion or another, so a host response is to detect the lack of class I molecules by inhibitory NK cell receptors on NK cells. Some viruses encode class I–like molecules as decoys to fool the NK cells with inhibitory receptors; one host counter-strategy is to evolve activating NK cell receptors that bind directly to the decoy molecules. In humans, killer immunoglobulin–like receptors (KIRs) recognize classical class I molecules, including some HLA-A alleles (A3 and A11), some HLA-B alleles (those with Bw4 epitopes) and all HLA-C alleles (divided into C1 and C2 allelic lineages). On most human cell types, HLA-A and HLA-B molecules are much better expressed than HLA-C molecules (Djaoud and Parham 2020). In chickens, the classical class I molecules encoded by the BF2 gene are far better expressed than those expressed by the BF1 gene (Shaw et al. 2007; Wallny et al. 2006). Chicken immunoglobulin-like receptor (ChIR) genes, some of which are very much like inhibitory and activating KIRs (along with others whose gene products bind the immunoglobulin IgY), are found on chromosome 31 in a region considered to be the equivalent of the leukocyte receptor complex (LRC) (Jansen et al. 2016; Laun et al. 2006; Lochner et al. 2010; Straub et al. 2013; Viertlboeck et al. 2005, 2009). There are not many studies examining either CTLs or NK cells in chickens; the limited data suggests that BF2 molecules are generally CTL ligands while BF1 molecules are NK ligands (Kim et al. 2018).

Another set of NK receptor genes are based on extracellular lectin-like domains, including the NKPR1 genes (also known as KLRB1 or CD161), encoded in the NKC. In humans, mice and rats, the NKRP1 genes are located next to (or near) the genes that encode lectin-like ligands: A single NKRP1 gene is next to a single LLT1 gene in humans, while Nkrp1/clr gene pairs are found in mice and rats, with variation driven by immune evasion with at least one virus (Bialoszewska and Malejczyk 2018; Kirkham and Carlyle 2014; Voigt et al. 2007). In chickens, the ortholog of NKRP1 is BNK, which sits next to the LLT1/clr homolog Blec (Kaufman et al. 1999a; Rogers et al. 2005) (with BNK and Blec sometimes referred to as Blec2 and Blec1, respectively, by some bioinformaticians, Shiina et al. 2007). In addition, other genes similar to Blec are found in the BG region and in the Y region (Rogers et al. 2003; Salomonsen et al. 2014) (later confusingly referred to by some bioinformaticians as Blec3 and so on, such that Blec2 in this nomenclature is structurally quite different from Blec1, Blec3 and so on, Shiina et al. 2007). BNK is highly polymorphic, while Blec is monomorphic (Rogers and Kaufman 2008). Contrary to expectations, the BNK molecule from a particular MHC haplotype was not stimulated by Blec (or by BF1 or BF2) molecules; instead, a protease-sensitive ligand was detected on spleen cells from young chickens (Viertlboeck et al. 2008). Whether different members of the Blec family are recognised by different BNK alleles, and whether Blec molecules are protease-sensitive has not yet been determined. As mentioned above, NKRP1-ligand gene pairs are found in the mammalian NKC; the two lectin-like genes found in the syntenic region in the chicken NKC were found to likely have different functions (Chiang et al. 2007; Neulen and Gȍbel 2012). The presence of the BNK-Blec gene pair was suggested as evidence that NKRP1-ligand gene pairs were originally in the MHC and translocated elsewhere during evolution (Rogers et al. 2005). Interestingly, some passerine birds have the BNK-Blec gene pair on the Z chromosome (Ekblom et al. 2011; Rogers and Kaufman 2016), so these genes may translocate with some facility, for reasons as yet unknown.

A single C4 gene marks the start of the class III region of the chicken MHC (Kaufman et al. 1999a, b), but isolation of the C4 protein in chicken blood revealed a completely different N-terminal sequence (Y. Palarasah, J. Kaufman and K Skjødt, unpublished). The cDNAs of both genes were cloned and sequenced, and used to map the second blood C4 to chicken chromosome 1. The two sequences were very different, but the single amino acid position that determines the specificity of thioester reactivity showed that the non-MHC C4 is the equivalent of C4A in mammals and the MHC C4 is the equivalent of C4B; these preferences were confirmed by biochemical assays. Specific monoclonal antibodies were derived and used to show that both genes are expressed in the liver, but that the non-MHC C4 was much better expressed in blood than the MHC C4, for reasons that are still being examined. Upon the first sequence of the chicken genome, the genes around the non-MHC C4 were found to define a syntenic region which was conserved at least to bony fish, with a similar gene in various vertebrates but lacking in mammals (Y. Palarasah, J. Kaufman and K Skjødt, unpublished). Strikingly, the C4A and C4B genes of humans have long been known to be extremely similar and likely the product of a recent duplication, leading to the evolutionary scenario that the genomes in the lineage leading to placental mammals lost the non-MHC C4 gene, duplicated the MHC C4 gene and then selected for the one copy to reproduce the enzymatic specificity of the lost non-MHC C4 gene. As this work was ongoing, another group of bioinformaticians came to similar conclusions about the evolutionary history (Nonaka et al. 2017).

Mammalian C4 molecules have long been known to have central roles in innate immunity, through cell lysis, through opsonisation of bacteria and fungi by macrophages and other myeloloid cells, and through attraction of certain cell types through an anaphylotoxin. However, the C4 genes have been found to exhibit variation that affects not only innate immunity, but also other physiological processes such as autoimmunity and synaptic pruning in the central nervous system (Johnson and Stevens 2018; Wang and Liu 2021). Indeed, single nucleotide polymorphisms (SNPs) in and around the C4 gene are among the top hits in genome-wide association studies (GWAS) with schizophrenia and other mental conditions, as well as sex-linked autoimmunity (Kamitaki et al. 2020; Sekar et al. 2016). The polymorphism of the chicken C4 gene and possible importance in various physiological functions has yet to be investigated.

Although the boundary of the opposite end of the BF/BL region has not been agreed upon, there is a single BG1 gene present along with an authentic butyrophilin gene (Kaufman et al. 1999a, b). Butyrophilin (and butyrophilin-like) genes in the MHC of humans and mice encode chains of heterodimers that play central roles in the maturation, tissue distribution and stimulation of γδ T cells (Di Marco Barros et al. 2016; Jandke et al. 2020; Ventourout et al. 2018). Of course, γδ T cells are created as part of the adaptive immune system, but like NKT cells with semi-invariant T cell receptors, some γδ T cells act in an innate manner, with the name “adaptate” (rather than “inaptive”) being coined to name this usage (Hayday 2019; Hayday and Vantourout 2020). In any case, there are only two butyrophilin genes known to be present in the chicken genome, one on chromosome 28 where it is known as Tvc, a receptor for avian leucosis virus (ALV) subtype C, and the other at the edge of the BF/BL region, with unknown function (Elleder et al. 2005; Kaufman et al. 1999). It has long been speculated that BG genes, which have some features in common with butyrophilins, might encode disulphide-linked dimers on the cell surface that play roles for γδ T cells similar to butyrophilins in mammals. Both the extracellular V domain and the long cytoplasmic tail formed of heptad repeats in BG molecules are polymorphic, but only the variation in the cytoplasmic tail seems to have been selected (Chattaway et al. 2016; Chen et al. 2018). A coiled-coil protein that affects actin-myosin interactions has been shown to originate in the cytoplasmic tail of a BG gene (Bikle et al. 1996). Also, a recombination event in the cytoplasmic tail of the BG1 gene has been identified and proposed to have a significant effect on resistance to tumours induced by Marek’s disease virus (Goto et al. 2009). Whether the BG1 chain might form a heterodimer with the chains encoded by the other BG genes in the BG region is not yet clear.

## Innate immunity genes in other regions on chicken chromosome 16

Outside of the BF/BL region is a region with many TRIM genes and then a region of many BG genes, and on the same chromosome is the Y region with at least one expressed non-classical class I gene, some non-polymorphic class II B genes and lectin-like receptor genes, followed by regions with olfactory receptors and scavenger receptors. Some of these genes are likely to be involved in innate immunity.

In mammals, there are at least 60 TRIM genes that function as E3 ligases involved in conjugation of substrates with ubiquitin, small ubiquitin-like modifier (SUMO) or interferon-stimulated protein of 15 kDa (ISG15). TRIM proteins are important for cell cycle, autophagy, development, cancer and autoimmunity (Di Rienzo et al. 2020; Hatakeyama 2017; Ozato et al. 2008). TRIM genes are well-known for acting as viral restriction factors at many levels including uncoating (TRIM5α), transcription (TRIM11, 32) and assembly (TRIM15, 22), and in regulation of interferon signalling (TRIM8), regulation of pattern recognition receptor signalling (TRIM25, 26, 27, 30α, 31, 38, 39 and 40) (Ozato et al. 2008) and mediating intracellular immunity directed by cytoplasmic IgG (TRIM21) (Bottermann and James 2018). All TRIM proteins have the tripartite motif: an N-terminal RING box for interaction with E2 liganses, one or two B-box domains and a coiled-coil region for oligomerisation, followed by various other domains, with most having a PRY-SPRY (also called B30.2) domain for ligand binding (Ozato et al. 2008).

The human MHC has 11 TRIM genes in the class I region, nine in a cluster (TRIM10, 15, 26, 31, 39, 40 and RNF39) and two singletons (TRIM27 and 38). The cluster of TRIM genes is found in the mouse MHC, but Trim27 and Trim38 are located on a different mouse chromosome. The human and mouse genes have some polymorphism, leading to association with various immune responses and autoimmune conditions (Jia et al. 2021). The chicken TRIM region has seven TRIM genes with PRY-SPRY domains, two each identified as having the most similarity to TRIM7, 27 and 39, and one orthologous to TRIM41, as well as three other Zn finger–containing genes (Ruby et al 2005; Shiina et al. 2007). TRIM7 and TRIM41 in the chicken MHC are located next to a guanine nucleotide binding protein homologous to GNB2L1; these three genes are located on human chromosome 5 (Guillemot et al. 1989; Ruby et al 2005). Nothing is yet known about the polymorphism or function of any of these chicken TRIM genes. The comparison of the human, mouse and chicken genomes underscores the fact that the TRIM genes come and go from the MHC syntenic region during evolution.

Next to the TRIM region is the BG region, which has many BG genes as well as a few pairs of a kinesin-like motor protein gene and a lectin-like gene similar to Blec (Salomonsen et al. 2014). As mentioned above, BG genes were discovered as erythrocyte cell surface antigens, but were then found to be expressed on lymphocytes and epithelial cells (Miller et al 1990; Salomonsen et al. 1991, 2014). As members of the B7 gene superfamily, it has long been speculated (Henry et al. 1999) that BG molecules act as their closest relatives in mammals, the butyrophilins and butyrophilin-like molecules, acting as co-stimulators and co-inhibitors for αβ and γδ T cells, and more recently as major regulators of location and activation of γδ T cells in an adaptate manner (Hayday 2019; Arnett and Viney 2014; Hayday and Ventourout 2020; Rhodes et al. 2016).

However, there are major differences in the domain organisation of butyrophilin, butyrophilin-like and BG molecules. The butyrophilin molecules have an extracellular immunoglobulin-like (Ig-like) V domain followed by an Ig-like C2 domain (with V-C2-V-C2 for butyrophilin-like molecules), followed by a transmembrane region, a short region of heptad repeats and a PRY-SPRY (B30.2) domain (Arnett and Viney 2014; Rhodes et al. 2016). In contrast, BG molecules are disulphide-linked dimers of chains with a single Ig-like V domain, a transmembrane region and a very long region of heptad repeats which presumably form a coiled-coil (Kaufman et al. 1990; Miller et al. 1991; Salomonsen et al. 2014). Although most butyrophilins are heterodimers, it is not yet clear whether BG molecules expressed on cells are homodimers or heterodimers, and if the latter, heterodimers of which gene products. Whether additional proteins (for example, orphan B30.2 domains) bind to the intracellular tail to provide function and whether BG molecules are recognised by T cells are both unclear.

An additional challenge is to understand the diversity of the BG genes, which exhibit much copy number variation (CNV) as well as sequence polymorphism selected in the intracellular cytoplasmic tails. While there are two stable singleton BG genes, BG1 in the MHC and BG0 on another chromosome, the BG genes in the BG region are arranged tandemly in the same transcriptional orientation, an arrangement ideal for recombination and deletion leading to expansion and contraction of the BG multigene family, manifested as CNV (Salomonsen et al. 2014). Although there is sequence variation throughout each BG gene, comparison of apparent alleles (that is, BG genes expressed in a particular cell type) suggests that selection for diversity is found only in the intracellular coiled-coils (Chattaway et al. 2016; Chen et al. 2018). This is even more surprising given that the promoter at the 5′ end of the gene determines in which cells a particular BG chain is expressed, but recombination leads to hybrids of the extracellular and intracellular parts of the protein (Salomonsen et al. 2014).

The Y locus is located on chicken chromosome 16, although a region of repeats leads to sufficient recombination that it is genetically unlinked to the B locus, as though they were on different chromosomes. The Y region has one or more class I genes encoding polymorphic YF chains, which bind β_2_m and have hydrophobic binding sites (Afanassieff et al. 2001; Hee et al. 2010). In mammals, the monomorphic CD1 and MR1 molecules also bind β_2_m and have binding sites for hydrophobic lipid tails and for bacterial metabolites, respectively (D’Souza et al. 2019; Ogg et al. 2019). The sequences of YF genes are not particularly like either CD1 or MR1. Recently, evidence has been presented that the Y locus is highly repetitive with many YF genes (Zhang et al. 2020). What kind of T cell recognises YF molecules and for what purpose is also a mystery. Also in the Y region are a variable number of class II B genes, apparently monomorphic and related to the polymorphic BLB genes in the chicken MHC, and some lectin-like genes related to Blec, which might be ligands for BNK.

Beyond the Y locus are regions of olfactory receptors and cysteine-rich domain scavenger receptors, as defined by cytogenetics and BAC sequences (Delany et al 2009; Miller et al 2014; Warren et al 2017). There is a huge superfamily of scavenger receptors in mammals that have many functions, including as pattern recognition receptors involved in innate immunity (Canton et al. 2013; Yu et al 2015). Many of the cysteine-rich domain scavenger receptors on chicken chromosome 16 are reported to have immunotyrosine-based inhibitory motifs (ITIMs) that are important for signalling (Miller et al 2014). Moreover, some ChIR genes are reported also to be in this region (Miller et al. 2014), although most are located in the LRC on chromosome 31 (Straub et al. 2013).

## Conclusions

Although the main focus of research on the BF/BL region of the B locus, the chicken MHC, has been those genes involved in adaptive immunity, there is a long list of genes actually or potentially important in innate immunity, or in those immune responses that combine features of innate immunity within the adaptive system, called by some adaptate. It appears that the simplicity of the adaptive immune system genes on this chicken microchromosome contrasts with the complexity of the genes potentially involved in innate and adaptate immunity. This contrast may be typical for non-mammalian vertebrates, where a single or a dominantly expressed classical class I molecule compares to many non-classical class I and innate immune genes (Kaufman 2018; Ohta et al. 2006; Flajnik et al. 1993; Grimholt et al. 2015; Langavin et al. 2019; Ohta et al. 2006).

A few seminal studies, such as the importance of the BG1 locus for resistance to Marek’s disease (Goto et al. 2009) and the identification of BF1 as an NK ligand (Kim et al. 2018), have provided enlightenment about function beyond just describing the molecular structures at the genomic and protein levels. Otherwise, there are mysteries everywhere to be understood, and the next years may be exciting, as genetic techniques are used to relate infection outcomes to genomic features. Among the more general approaches, the development of SNP typing methods (particularly those that cover the TRIM region, Fulton et al. 2016) may help link structure to function for such innate and adaptate genes. It will be more difficult in the BF/BL region due to the low level of recombination and in the BG region due to the rampant CNV, so there is much work but also potentially exciting results ahead.
